# Disaggregate level estimates and spatial mapping of food insecurity in Bangladesh by linking survey and census data

**DOI:** 10.1371/journal.pone.0230906

**Published:** 2020-04-10

**Authors:** Md. Jamal Hossain, Sumonkanti Das, Hukum Chandra, Mohammad Amirul Islam

**Affiliations:** 1 Bangladesh Agricultural University, Mymensingh, Bangladesh; 2 University of Southampton, Southampton, United Kingdom; 3 Shahjalal University of Science & Technology, Sylhet, Bangladesh; 4 Maastricht University, Maastricht, The Netherlands; 5 ICAR-Indian Agricultural Statistics Research Institute, PUSA, New Delhi, India; 6 Bangladesh Agricultural University, Mymensingh, Bangladesh; Universiteit Gent, BELGIUM

## Abstract

Food insecurity is an important and persistent social issue in Bangladesh. Existing data based on socio-economic surveys produce divisional and nationally representative food insecurity estimates but these surveys cannot be used directly to generate reliable district level estimates. We deliberate small area estimation (SAE) approach for estimating the food insecurity status at district level in Bangladesh by combining Household Income and Expenditure Survey 2010 with the Bangladesh Population and Housing Census 2011. The food insecurity prevalence, gap and severity status have been determined based on per capita calorie intake with a threshold of 2122 kcal per day, as specified by the Bangladesh Bureau of Statistics.The results show that the food insecurity estimates generated from SAE are precise and representative of the spatial heterogeneity in the socioeconomic conditions than do the direct estimates. The maps showing the food insecurity indicators by district indicate that a number of districts in northern and southern parts are more vulnerable in terms of all indicators. These maps will guide the government, international organizations, policymakers and development partners for efficient resource allocation.

## Introduction

Achieving food security for all remains one of the major development goals throughout the world. Food security exists when all people, at all times, have physical and economic access to sufficient, safe and nutritious food that meets their dietary needs and food preferences for an active and healthy life as described by Food and Agriculture Organization (FAO) of the United Nations [1]. Inversely food insecurity exists when people do not have adequate physical, social or economic access to food. The food security is one of the highest priorities of the Government of Bangladesh to achieve the second Sustainable Development Goal (SDG 2). Although the food security situation in Bangladesh has improved during the last few decades, growing inequality among lower administrative boundary levels is a serious concern. Policy interventions and foreign aids are more likely to be effective in reducing spatial inequality of food security if resources can be allocated and distributed based on local level food insecurity status. Despite the high importance, the estimates of food insecurity indicators at local area or lower administrative boundary (e.g. district) level are still lacking.

The Bangladesh Bureau of Statistics (BBS) uses the estimate of food insecurity indicators as an alternative of poverty indicators calculated from the Household Income and Expenditure Survey (HIES) of BBS, see for example, BBS and UNWFP [2]. In Bangladesh “food insecurity” is defined as an average intake of less than 2122 kcal per capita per day based on the Direct Calorie Intake (DCI) method. However, the district level estimates of food insecurity indicators (as an alternative of poverty indicators) calculated by the BBS based on HIES 2000 data [2] are now obsolete in terms of its use-effectiveness. Thus, the district level estimates of food insecurity indicators using the latest round of HIES 2010 data can provide invaluable information for the policy planners to formulate effective action plans to achieve the relevant SDGs. Hence, to fill in the gaps in this respect, this paper aims to generate the district level estimates of food insecurity indicators using Empirical Best Prediction (EBP) approach of Small Area Estimation (SAE), seemingly reasonably well suited method for Bangladesh context (discussed in section 2), with supplementary interactive maps.

The rest of the paper is organized as follows. Section 2 delineates an overview of SAE methods to search for a better option to estimate food insecurity and describes the data from the HIES 2010 and the Bangladesh Population and Housing Census 2011 (hereafter Census 2011). This Section also provides discussion on model specification required for the SAE analysis. Section 3 briefly describes SAE methodology, in particular the EBP, employed for generating the district level estimates of the food insecurity indicators. Application of the EBP to food insecurity mapping at the district level is presented in Section 4. Finally, Section 5 set outs the main conclusions and recommendations.

### Overview of SAE approach: Searching a better option

The existing data, based on national level socio-economic survey (e.g. HIES conducted by the BBS), produce estimates that are representative of the macro-geographical units (e.g. national and division (admin-1)) and cannot be used directly to produce reliable micro or disaggregate or local level (also referred as small area e.g. district or sub-district level) statistics due to very small or even zero sample sizes. In the survey literature, an area is regarded as small if the area-specific sample size is not large enough to ensure that a direct survey estimator has adequate precision. In this context, the statistical methodology that tackles this problem of small sample sizes is often referred as SAE theory in the survey literature [3, 4]. The technique is a model-based method that links the variable of interest from survey with the auxiliary information available from other data sources (e.g. census) for small areas. See Rao and Molina [3] for a comprehensive overview of SAE techniques.

The standard approach of SAE methods based on both unit-level and area-level modelling have been developed comprehensively for linear parameters such as means and totals. However, direct use of such SAE methods to produce estimates for non-linear and complex parameters, for instance Foster-Greer-Thorbecke (FGT) type food insecurity and poverty indicators becomes problematic [5] and appropriate transformation of the target variable is needed (see methodology section for details). As unit (i.e. household) level data is accessible, this paper focuses on unit level small area modeling and considers the SAE of food insecurity parameters. In this context, the three commonly used SAE methods based on unit-level model are the Elbers, Lanjouw and Lanjouw (ELL) or World Bank method [6], the empirical Bayes (Best) prediction (EBP) method [7] and the M-Quantile (MQ) method [8]. What follows, these methods are compared to suggest for a better approach for estimating food insecurity indicators.

The ELL method is the simplest one in terms of application and theoretical complexity. In the ELL method, the original/transformed values of the target response is assumed to follow a unit-level nested error regression model where households are nested within the cluster that is clusters instead of the target small areas are assumed to have random effects. Although the ELL method is an attractive and the easiest one to implement, it provides poor mean squared errors (MSE) even if the bias is usually small [9]. Since the ELL method assumes cluster-variability rather than area-variability, the ELL estimator performs poorly and can even perform inferior to direct estimator when unexplained between-area variation remains in the model after accounting a number of explanatory variables [7]. Moreover, this method is not optimal for a given distribution since it does not give the empirical best estimator [9]. In the MQ method, the between-area variations are captured by calculating area-specific M-quantile coefficients instead of random effects. The distinguishing features of this method are distribution free assumptions on the model errors and the area effects, and this also allows outlier robust inference. However, when the functional form of the relationship between the q^th^ MQ and the covariates is non-linear, it can lead to biased estimates [10]. The EBP method is based on the assumption that a suitable transformation (e.g. log) of the response variable will follow a nested-error linear regression model [11], called hereafter BHF (Battese-Harter-Fuller) model, with normally distributed errors. The EBP is similar to the ELL method but generates census predictions of response variable such as per capita calorie intake by using the conditional predictive distribution of the out-of-sample data, given the sample data under the BHF model. This method gives the best predictions by minimizing the MSE under the assumed unit-level model. Further, the EBP performs better than the ELL method when underlying assumptions are satisfied and the unexplained between-area variation remains significant [12].

The performances of three methods (i.e. ELL, EBP and MQ) differ in terms of their underlying model assumptions particularly distinctions in the consideration of random effects [13]. One method performs better when the real dataset satisfies the respective underlying model assumptions. The EBP utilizes the survey data to narrow down the random area effects while the conventional ELL method makes no such attempt. Consequently, the EBP method will make a difference for areas with some information in the survey, while the EBP method reduces to the conventional ELL prediction for those areas without any information in the survey. For Bangladesh poverty mapping study, when the prediction level goes down to sub-districts from district, there are many small domains without any information in the survey data. To use the survey information, the EBP method has been added in the World Bank POVMAP software version-2.5 [14] with the note that the conventional EBP method based prediction is expected to do better if there are relatively large random area effects, if many of the small areas are covered by the survey, while the error distributions can be reasonably well approximated by a normal distribution. In our analysis, the target variable of interest is daily per capita calorie intake averaged at the household level and on logarithmic (log) scale is approximately normally distributed with a negligible number of influential observation and the random district specific effect is significant for this data (for detail results see next Section). Further, all the target small domains (here district) are covered in the survey data. As a consequence, following [14] this paper adopts the EBP method rather than the ELL and the MQ methods to generate the district level estimates of the food insecurity indicators in Bangladesh by linking the HIES 2010 and the Census 2011.

## Data and model specification

In this study, the variable of interest for which small area estimates are required is taken from the HIES 2010 conducted by BBS. The HIES 2010 survey data is collected following a two-stage stratified sampling design covering all the 7 divisions and 64 districts. Detail sampling plan is available in the HIES report [15]. In HIES 2010, the district-wise sample size ranges from 120 to 480 with an average of 191. The sampling fraction varies from 0.00019 to 0.00052 across district with an average of 0.00040. Eventually, the district level sample sizes are not sufficient to produce reliable estimates of the food insecurity indicators and their associated standard errors at district level. The application of SAE technique is an obvious choice for obtaining the district level estimates of the food insecurity indicators. The daily per capita calorie intake averaged at the household level is used as a response variable to estimate the food insecurity indicators. The per capita calorie intake is slightly right skewed, and so the final response variable is prepared by taking log-transformation by adding up a fixed quantity to make it always positive as suggested by Molina and Marhuenda [16]. Thus, the log-scale daily per capita calorie intake is used as the response variable in the BHF model considered in this paper.

The auxiliary variables for this analysis are obtained from the Census 2011. Since the full census data set is not accessible, the permitted 5% Census 2011 data are used. As auxiliary variables, we used only those variables for modelling and predicting that are common and comparable between HIES 2010 and Census 2011. Also, some contextual variables at sub-district levels are created from the Census 2011 and combined with the HIES 2010. There are 70 auxiliary variables (covariates) scrutinized for fitting a 2-level BHF model considering household as the first level and district as the second level. We adopted the principle of hierarchical modelling technique for selecting the best set of covariates [17]. The two-way interaction terms are included in the model if only their corresponding main effects are also included. The interaction and non-linear terms are added carefully and judiciously. Following this approach, we used a stepwise regression between log-transform per capita calorie intakes and 70 different auxiliary variables to identify the significant covariates. Finally, 23 auxiliary variables that significantly explain the linear model with R-squared (R^2^) value of 22.5 percent, are identified for the use in the SAE analysis. Although diagnostic plots are not reported here, the residual diagnostic plots namely histogram and normal P-P plot of standardized residual for this data reveal fitted model satisfies the underlying assumption of normality and homoscedasticity reasonably well. Though the R^2^ value is not particularly high, it is reasonable to obtain precise disaggregate level estimates, if the unexplained variation is mostly at household level rather than area-level [18].

The summary results of the finally selected 2-level BHF model are given in Table 1. The estimates of fixed effect parameters along with their level of significance are shown in Table 2. The results in Table 1 clearly reveal that the significant contribution of random district specific effect $\left(\sigma_{u}^{2}\right)$ in the variability of response variable in the null model. The between district variation has been reduced when covariates are included in the null model. Following the idea of Nakagawa and Schielzeth [19], the marginal and conditional R-squared values for the full model indicate that about 22.2% of total variation is explained by the fixed effects alone and about 33.0% of total variation is explained by both the fixed and random effects. The smaller AIC value for full mixed-effects model also suggests the goodness of the fitted full model. The intra-class correlation coefficient (ICC) suggests that about 13% of the total variability in the response are due to between districts variation. The likelihood ratio (LR) test also confirms the significance of the between- district variation ($\chi_{\left(1\right)}^{2}=1416.46,$ P-value < 0.001).

**Table 1:** Summary statistics of the fitted 2-level (2L) linear mixed-effects model (BHF model) using REML method of estimation.

Model	DF	Marginal R^2^	Conditional R^2^	Random-effect Parameters		ICC*	AIC
				$\left(\sigma_{e}^{2}\right)$	$\left({\hat{\sigma }}_{u}^{2}\right)$		
2L: Null	3	-	0.116	0.0512	0.0067	0.1164	-1430.63
2L: Full	26	0.2219	0.326	0.0396	0.0061	0.1336	-4543.26

LR test vs. Linear model: $H_{0}:\sigma_{u}^{2}=0,\;\chi_{\left(1\right)}^{2}=1416.46,$ P-value = 0.000

*Intra-class correlation coefficient

**Table 2:** Estimate of fixed effect parameters along with their significance level of the fitted 2-level linear mixed-effects model (BHF model) using REML method of estimation.

Variables	Estimate	SE	z	p-value
hh size	-0.0380	0.0020	-19.0500	0.0000
hheads age	0.0006	0.0002	3.0900	0.0020
number of rooms in hh	0.0246	0.0019	13.0500	0.0000
hh located in rural area	0.0329	0.0104	3.1700	0.0020
hhead employed	0.0131	0.0055	2.3900	0.0170
hhead widowed	-0.0401	0.0075	-5.3500	0.0000
hhead divorced or separated	-0.0816	0.0193	-4.2200	0.0000
own house	0.0455	0.0092	4.9600	0.0000
rented house	0.0356	0.0104	3.4300	0.0010
pucka house	0.0315	0.0073	4.3200	0.0000
semi-pucka house	0.0171	0.0055	3.0800	0.0020
hhead has primary education	0.0220	0.0052	4.2400	0.0000
hhead has tertiary education	0.0131	0.0050	2.6000	0.0090
hh size squared	0.0024	0.0003	8.4500	0.0000
hh size in rural area	0.0068	0.0021	3.3000	0.0010
prop. of 15–59 yrs. persons in hh	0.2462	0.0122	20.2500	0.0000
prop. of 60+ yrs. persons in hh	0.1398	0.0171	8.1900	0.0000
prop. of 1–4 yrs. children in hh	-0.2564	0.0181	-14.1800	0.0000
prop. of 0 yr. children in hh	-0.3847	0.0342	-11.2600	0.0000
prop11-15yrs. female att.school	0.0839	0.0213	3.9400	0.0000
prop.11-15yrs. males att. school	0.1840	0.0207	8.9000	0.0000
Barisal division	-0.0934	0.0349	-2.6800	0.0070
Dhaka division	-0.0449	0.0230	-1.9600	0.0500
constant	7.5936	0.0211	360.1200	0.0000
$Wald\;:\;\chi_{\left(23\right)}^{2}$	3597.56			
Prob> $\chi_{\left(23\right)}^{2}$	0.000			
Number of district	64			
Log likelihood	2196.74			
Number of observations (HH)	12240			

**Fig 1:**
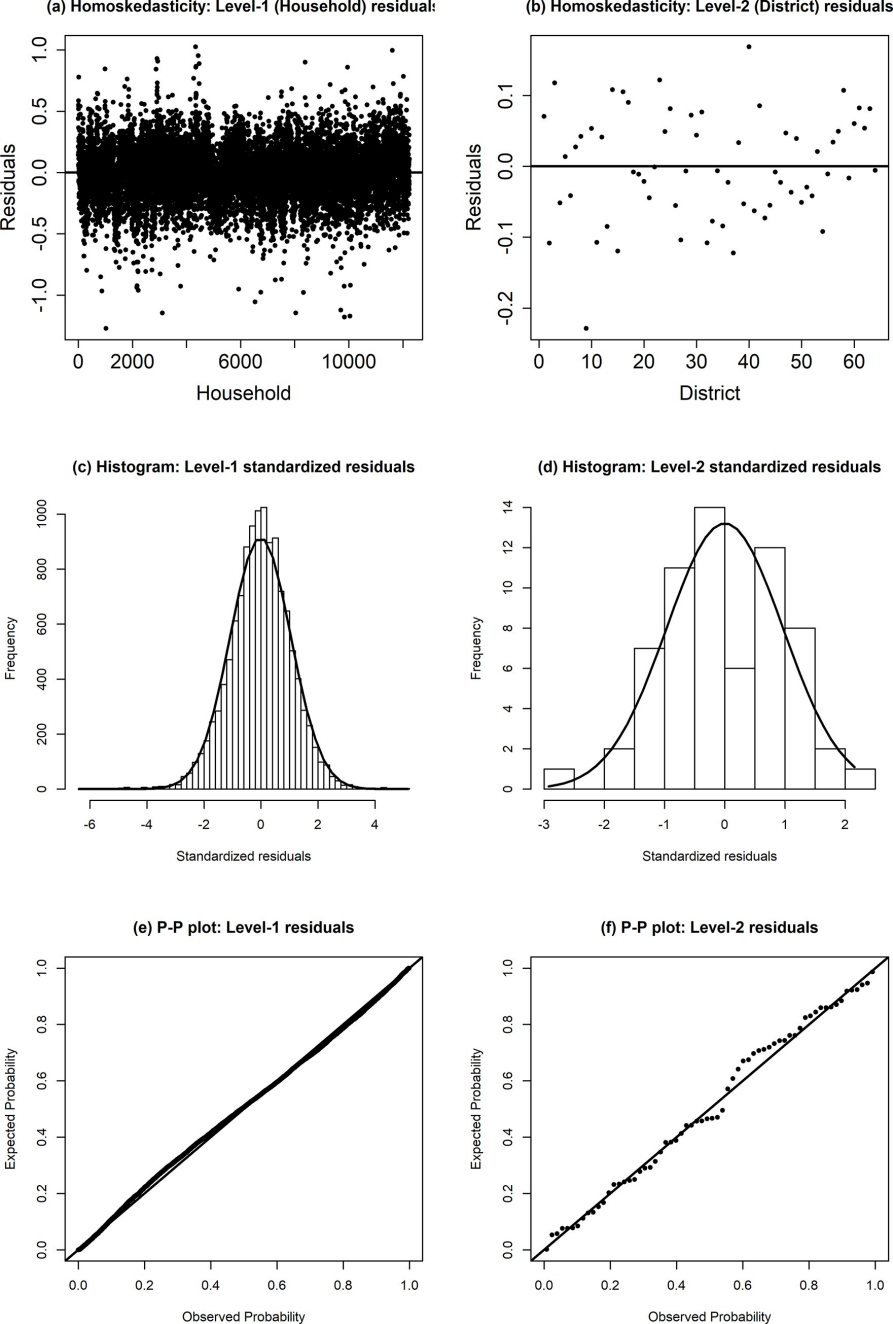
Distribution of residuals, histogram and normal p-p plot of residuals of the level 1 (left hand side) and level 2 (right hand side) obtained from the fitted 2-level linear mixed-effects model (BHF model).

The BHF model is based on distributional assumption of level 1 (household) and level 2 (district) random effects (residuals), i.e. both the level 1 and 2 random effects in model are independently and identically distributed values from normal distributions with mean zero and fixed variances. Standard diagnostics such as distribution of residuals, histograms and normal probability (p-p) plots are used for this purpose. Diagnostic plots of the level 1 and 2 residuals obtained from the fitted BHF model are shown in Fig 1. The plots in Fig 1 indicate that these distribution features hold for both level 1 and 2 residuals when fitted to HIES 2010 data. We also observed that the household as well as the district level residuals are randomly distributed, and that their line of best fit does not significantly differ from the line *y* = 0 in both cases. Model diagnostics are therefore satisfactory for both the BHF model fitted to HIES 2010 data. The EBP method of SAE is therefore expected to provide efficient estimates of district level food insecurity indicators obtained from the fitted BHF model.

## SAE Methodology: EBP method

This Section briefly presents an overview of SAE method used in the estimation of district wise food insecurity indicators. To start, let us assume that there is a known number *N*_*i*_ of population units in area *i*, with *n*_*i*_ of these sampled. The total number of units in the population is $N=\sum_{i=1}^{D}N_{i}$, with corresponding total sample size $n=\sum_{i=1}^{D}n_{i}$. We use *s* to denote the collection of units in sample, with *s*_*i*_ the subset drawn from small area *i* (i.e. |*s*_*i*_| = *n*_*i*_), and use expressions like *j*∈*i* and *j*∈*s* to refer to the units making up area *i* and sample *s* respectively. Similarly, *r*_*i*_ denotes the set of units in area *i* that are not in sample, with |*r*_*i*_| = *N*_*i*_−*n*_*i*_ and *U*_*i*_ = *s*_*i*_∪*r*_*i*_. Let, *E*_*ij*_ denotes the value of the variable of interest e.g., per capita calorie intake of household *j*(*j* = 1,…,*N*_*i*_) in district *i* (*i* = 1,…,*D*). The quantity of interest is the small area food insecurity indicator *F*_*αi*_, followed from Foster et al. [5], is defined as

$$F_{\alpha i}=N_{i}^{-1}\sum_{j\in U_{i}}F_{\alpha ij},$$ (1)

where *F*_*αij*_ = (1−*E*_*ij*_/*k*)^*α*^*I*(*E*_*ij*_≤*k*) and *k* is a preset food insecurity line (i.e. *k* = 2122 kcal). The food insecurity indicators (hereafter FGT) are referred as food insecurity prevalence (FIP), Food insecurity gap (FIG) and Food insecurity severity (FIS) when *α* = 0, 1 and 2 respectively. With this, our aim is to make inference about the FGT food insecurity indicators *F*_*αi*_ for small area *i*. The design-based direct estimator (Direct) for the FGT food insecurity indicator *F*_*αi*_ is

$${\hat{F}}_{\alpha i}^{Dir}=\sum_{j\in s_{i}}w_{ij}F_{\alpha ij},i=1,\ldots ,D;\alpha =0,1,2.$$ (2)

Here $w_{ij}=w_{ij}^{*}/\sum_{j\in s_{i}}w_{ij}^{*}$ is normalized survey weights and $w_{ij}^{*}$ is inverse of the inclusion probability for unit *j* in area *i*. The design-based variance of the direct estimator ${\hat{F}}_{\alpha i}^{Dir}$ can be approximated by,

$$var({\hat{F}}_{\alpha i}^{Dir})\approx {\sum_{j\in s_{i}}w_{ij}(w_{ij}-1)(F_{\alpha ij}-{\hat{F}}_{\alpha i}^{Dir})}^{2}.$$ (3)

The direct estimator becomes inefficient when area specific sample size is small and further, for areas with no sample data, direct estimates cannot be used. In this context, EBP method of Molina and Rao [7] is often used for estimating the FGT indicators [7]. Let **x**_*ij*_ be the vector of values of *p* unit level auxiliary variables associated with the target variable *y*_*ij*_ = log(*E*_*ij*_). A two-level nested error model (which is a special case of linear mixed model), often referred as BHF model in SAE [11], considering household at level-1 and target small area (here districts) at level-2 is

$$y_{ij}=x_{ij}^{T}\beta +u_{i}+e_{ij},\;j=1,\ldots ,N_{i};\;i=1,\ldots ,D.$$ (4)

Here **β** is a *p-*vector of fixed effects parameter; $u_{i}∼N(0,\sigma_{u}^{2})$ is a random area specific effect associated with area *i* and $e_{ij}∼N(0,\sigma_{e}^{2})$ is an individual level random effect for unit *j* in area and assume that they are mutually independent. The FGT food insecurity indicator for small area *i* given by (1) can be expressed as

$$F_{\alpha i}=N_{i}^{-1}\left\{\sum_{j\in s_{i}}F_{\alpha ij}+\sum_{j\in r_{i}}F_{\alpha ij}\right\}.$$ (5)

where *s*_*i*_ and *r*_*i*_ denote sample and non-sample vectors of size *n*_*i*_ and *N*_*i*_−*n*_*i*_ units of area *i*.

Molina and Rao [7] obtained the empirical best predictor (EBP) of *F*_*αij*_ = *h*_*α*_(*y*_*ij*_), as a non-linear function of *y*_*ij*_,by minimizing the MSE without restrictions of linearity or unbiasedness and is given by

$${\hat{F}}_{\alpha ij}^{EBP}=E_{y_{r_{i}}}[h_{\alpha }(y_{ij})|y_{s_{i}}]=\int h_{\alpha }(y_{ij})f_{y_{j}}(y_{ij}|y_{s_{i}})dy_{ij},j\in r_{i},$$ (6)

where $f_{y_{j}}(y_{j}|y_{s_{i}})$ is the conditional density of $y_{r_{i}}$ given the sample data $y_{s_{i}}$. The expected value in (6) cannot be calculated explicitly due to the complex non-linear parameters of *F*_*αij*_ = *h*_*α*_(*y*_*ij*_), even if this conditional distribution was completely known. In this case, Molina and Rao [7] proposed to estimate the unknown model parameters by consistent estimators such as Maximum Likelihood (ML) or the Restricted Maximum Likelihood (REML) estimators $\hat{\theta }={\left({\hat{\sigma }}_{u}^{2},{\hat{\sigma }}_{e}^{2}\right)}^{T}$ of θ, and then obtaining the Empirical Bayes estimator of *F*_*αij*_ by a Monte Carlo approximation of the expected value in (6). The steps of the estimation procedure are:

- Generate out-of-sample vectors of $y_{ij}^{\left(l\right)},\;l=1,\ldots ,L,j\in r_{i}$ for large *L*, from the estimated conditional distribution $f_{y_{ij}}(y_{r_{i}}|y_{s_{i}};\hat{\beta },\hat{\theta })$
- Calculate the target quantity $F_{\alpha ij}^{\left(l\right)}=\sum_{j}h_{\alpha }(y_{ij}^{\left(l\right)})$ for each *l* = 1,…,*L* by combining sampled *y*_*ij*_,*j*∈*s*_*i*_ and non-sampled $y_{ij}^{\left(l\right)},j\in r_{i}$
- Average the target quantity over the *L* simulations as

$${\hat{F}}_{\alpha ij}^{EBP}=\frac{1}{L}\sum_{l=1}^{L}h_{\alpha }(y_{ij}^{\left(l\right)}),\;j\in r_{i}.$$ (7)

Since the size of $y_{r_{i}}$ is typically very large, generation of $y_{r_{i}}$ might be computationally cumbersome from a multivariate distribution and so Molina and Rao [7] proposes to generate $y_{r_{i}}$ from univariate distribution as

$$y_{{}_{ij}}^{\left(l\right)}=x_{ij}^{T}\hat{\beta }+{\hat{u}}_{i}+v_{i}+\varepsilon_{ij},\;j\in r_{i},\;i=1,\ldots ,D,$$ (8)

with $v_{i}∼N(0,{\hat{\sigma }}_{u}^{2}(1-{\hat{\gamma }}_{i}))$, $\varepsilon_{ij}∼N(0,{\hat{\sigma }}_{e}^{2})$ and ${\hat{\gamma }}_{i}=\sigma_{u}^{2}/\left(\sigma_{u}^{2}+n_{i}^{-1}\sigma_{e}^{2}\right)$. The EBP of the food insecurity measure *F*_*αi*_ is then given by

$${\hat{F}}_{\alpha i}^{EBP}=N_{i}^{-1}\left\{\sum_{j\in s_{i}}F_{\alpha ij}+\sum_{j\in r_{i}}{\hat{F}}_{\alpha ij}^{EBP}\right\}.$$ (9)

For areas with zero sample size, the EBP (9) reduces to a synthetic type estimator given by

$${\hat{F}}_{\alpha i}^{EBP}=N_{i}^{-1}\sum_{j\in r_{i}}{\hat{F}}_{\alpha ij}^{EBP}.$$ (10)

The MSE estimates are required to measure the precision of estimates and also to construct the confidence interval for the estimates. Following González-Manteiga et al. [20] and Molina and Rao [7], the MSE estimate of (9) is obtained using the parametric bootstrap method. The EBP (9) defined under model (4) and its associated parametric bootstrap MSE estimates can be obtained using *sae* package in R [16].

## Results and discussions

The district level estimates of three food insecurity indicators namely FIP, FIG, and FIS are generated from the EBP method under BHF model (4) with 23 significant covariates. The parametric bootstrap MSE estimates used in this analysis are based on *B* = 100 samples and *L* = 50 samples in the EBP estimation. In SAE application, two types of diagnostics measures are suggested and applied: (i) the model diagnostics, and (ii) the diagnostics for the small area estimates. The model diagnostics are applied to verify model assumptions. The other diagnostics are used to validate the reliability of the model-based small area estimates. In model (4), both level 1 (household level) and level 2 (district level) random effects (residuals), are assumed to be independently and identically distributed values from a normal distribution with mean zero and fixed variance. The standard diagnostics implemented reveals that the normality assumptions are satisfied reasonably well for the data utilized in this analysis.

To validate the reliability of the small area estimates produced by the EBP method (i.e. model-based small area estimates) from the fitted BHF model, a set of diagnostics is required. For example, model-based small area estimates should be consistent with unbiased direct estimates, and more precise than direct estimates. The values for the model-based small area estimates should be consistent with the unbiased direct estimates. Further, the model-based small area estimates should have MSEs significantly lower than the variances of the corresponding direct estimates [21, 22]. In this context, we deliberate three widely used diagnostics, viz. the bias diagnostics, coefficient of variation (CV) and 95% confidence intervals (CIs) for the small area estimates. In addition, we inspect the aggregation diagnostic where the model-based estimates are aggregated to higher level and compared with direct estimates at this level [21].

**Fig 2:**
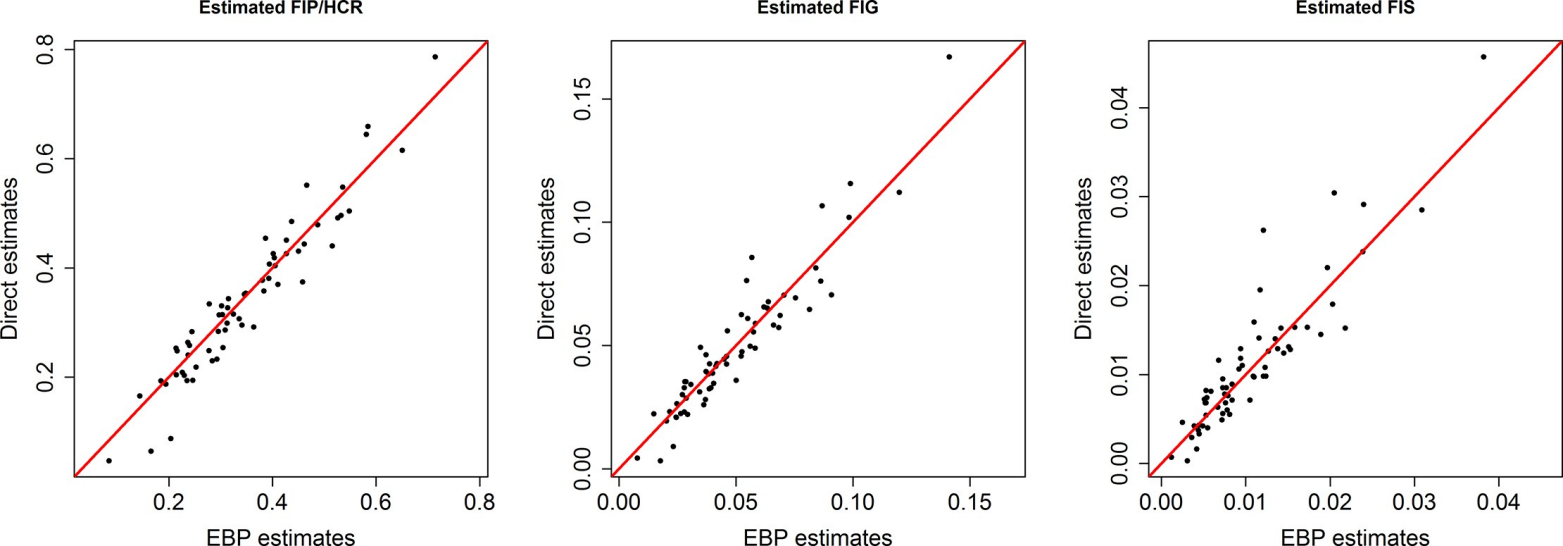
Bias diagnostics plots of FIP/HCR (left), FIG (centre), and FIS (right) food insecurity indicators generated by the EBP method with *y* = *x* line (solid) and regression line (dotted).

The bias diagnostics is applied to examine if the model-based small area estimates are less extreme when compared to the direct estimates. Further, if the direct estimates are unbiased, their regression on the true values should be linear and correspond to the identity line. Hence, if the small area estimates are also close to the true values the regression of the direct estimates on the model-based estimates should be similar. In this case, we plot direct estimates (here weighted direct survey estimates) on the vertical axis against the model-based estimates on the horizontal axis and looked for the divergence of the fitted regression line from *y* = *x* by testing for intercept = 0 and slope = 1 [21, 22]. The bias scatter plots of different food insecurity indicators are displayed in Fig 2. The plots in Fig 2 indicate that the model-based estimates are less extreme when compared to the direct estimates, demonstrating the typical SAE outcome of shrinking more extreme values towards the average. That is, the model-based small area estimates lie along the line *y* = *x* for most of the districts, which specifies that they are approximately design unbiased. The values of R^2^ for the fitted regression line between the direct estimates and the model-based estimates for three food insecurity indicators FIP/HCR, FIG, and FIS are 92, 89 and 83 per cent respectively. The calculated Pearson’s correlation coefficients between direct and the model-based estimates (0.96 for FIP/HCR, 0.94 for FIG and 0.91 for FIS) also suggest the consistency of the model-based estimates with the direct estimates.

We also use Goodness of fit (GoF) diagnostic. This tests whether the direct and model-based small area estimates are statistically different. The null hypothesis is that the direct and model-based small area estimates are statistically equivalent. The alternative is that the direct and model-based estimates are statistically different. The GoF diagnostic is computed using the Wald statistic for every model-based estimate:

$$W=\sum_{d}\left\{\frac{{\left(\mathrm{Direct}\;\mathrm{estimat}e_{d}‐\mathrm{Model}\;\mathrm{based}\;\mathrm{estimat}e_{d}\right)}^{2}}{var(\mathrm{Direct}\;\mathrm{estimat}e_{d})+mse(\mathrm{Model}\;\mathrm{based}\;\mathrm{estimat}e_{d})}\right\}.$$

The value from the test statistic is compared against the value from a chi-square distribution with *D = 64* degrees of freedom which is 83.675 at 5% level of significance. A small value (<83.675 in this case) indicates no statistically significant difference between model-based and direct estimates. The values of Wald statistic for the model-based estimates of FIP/HCR, FIG and FIS are 55.43, 67.72 and 70.34 respectively. These values are smaller than the 83.675, which reveal that model-based estimates are consistent with the direct estimates. Overall, the bias diagnostics show that the estimates generated by the model-based SAE method appears to be consistent with the direct estimates.

**Fig 3:**
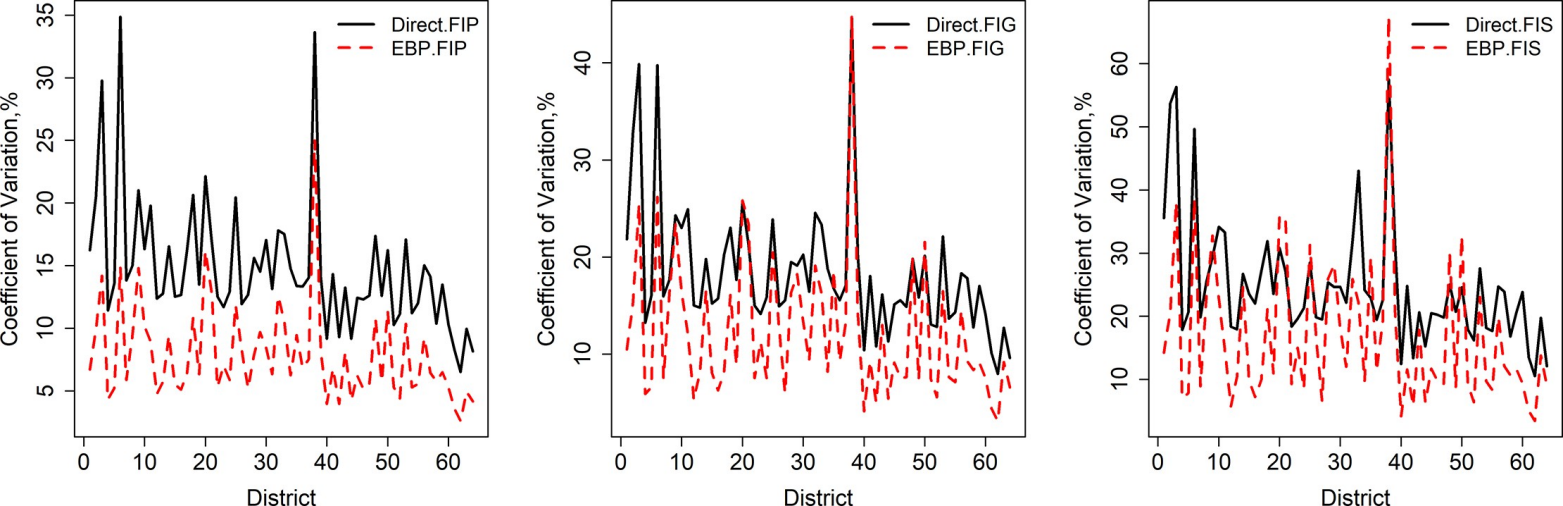
**District-wise percentage coefficient of variation (CV, %) of FIP/HCR (left), FIG (centre), and FIS (right) food insecurity indicators generated by direct and EBP method.** Districts are arranged in increasing order of sample size.

The percent CV is calculated to assess the improved precision of the model-based estimates generated by the EBP method compared to the direct estimates. The CV shows the sampling variability as a percentage of the estimate. Estimates with large CVs are considered unreliable. Fig 3 shows the district-wise values of percentage CV for direct (Direct) and EBP methods. The estimates of food insecurity indicators (FIP/HCR, FIG, and FIS) in Bangladesh by District obtained via the Direct and EBP methods along with their percentage CVs and and 95 confidence intervals are set out in S1–S3 Appendices. From the results presented in S1–S3 Appendices and shown in Fig 3, it is evident that the CVs of the direct estimates are slightly higher and therefore the estimates are unreliable. As expected, the larger CVs occur in the district smaller sample size. Though there is no exact role of thumb for the CV, 20% CV is maintained by the Office for National Statistics in the United Kingdom [23]. The CVs in Fig 3 show that the direct estimates of food insecurity prevalence (FIP/HCR) have CVs over 20% for several districts, whereas the CVs of the EBP estimates do not exceed this limit for any of the districts. The similar scenarios are also observed in case of FIG and FIS, except in few districts. Overall, the EBP estimates are more reliable than the direct estimates in terms of percentage CV for all the food insecurity indicators. The district-wise 95% CIs of the model-based and the direct estimates are reported in S1–S3 Appendices. In general, 95% CIs for the direct estimates are wider than the 95% CIs for the model-based estimates (see S1–S3 Appendices). The direct 95% CI estimates are wider due to large standard errors.

We also examine the aggregation of the model-based estimates of food insecurity prevalence at divisional and national level. Standardized differences between the two estimators (i.e. Direct and EBP) calculated $Z=\frac{\left(\mathrm{EBP}\;\mathrm{estimate}‐\mathrm{Direct}\;\mathrm{estimate}\right)}{\sqrt{var(\mathrm{Direct}\;\mathrm{estimate})+MSE(\mathrm{EBP}\;\mathrm{estimate})}}$ are shown in Table 3. The Z score is used to examine how the small area estimates differ from the design-unbiased direct estimates. The Z-scores are observed within three standard errors of the direct estimates, indicating a reasonable level of agreement between the two estimators. In all cases, except the estimate for the national level where the standard errors (SEs) are exactly equal, the small area estimates are more precise than the direct estimates. The improvement of SAE estimates in terms of SE is expectedly less at the division and national as expected due to sufficient sample size, however significant gains are expected at the lower administrative units (say, district or sub-district).

**Table 3:** Comparison of direct and EBP estimates of food insecurity prevalence (FIP/HCR) and their standardized difference.

Division	EBP		Direct		Z
	FIP/HCR	SE	FIP/HCR	SE	
Barisal	0.487	0.013	0.453	0.022	1.327
Chittagong	0.371	0.010	0.346	0.013	1.550
Dhaka	0.405	0.008	0.414	0.012	-0.646
Khulna	0.334	0.004	0.318	0.014	1.064
Rajshahi	0.310	0.006	0.288	0.014	1.405
Rangpur	0.276	0.008	0.262	0.015	0.831
Sylhet	0.299	0.016	0.288	0.019	0.447
Bangladesh	0.376	0.006	0.351	0.006	2.864

Summary statistics of the estimated food insecurity indicators for the 64 districts and along with their SEs generated by the Direct and the EBP methods are shown in Table 4. The results reveal that the averages of the EBP estimates of food insecurity indicators are slightly higher compared to those of the direct estimates but with a lower variation. As for example, average values of FIP/HCR are estimated by the direct and the EBP methods are 34.4% and 35.1% respectively. The standard deviations of these estimates generated by the direct and the EBP methods are 12.8% and14.2% respectively.

**Table 4:** Summary statistics of food insecurity indicators.

	Parameter	Method	Minimum	Maximum	Average	Standard Deviation
	FIP/HCR	Direct	0.047	0.786	0.344	0.142
		EBP	0.085	0.715	0.351	0.128
Estimate	FIG	Direct	0.0033	0.1670	0.0495	0.029
		EBP	0.0079	0.1412	0.0497	0.026
	FIS	Direct	0.0003	0.0457	0.0112	0.008
		EBP	0.0012	0.0382	0.0108	0.007
	FIP/HCR	Direct	0.0158	0.0815	0.0454	0.0120
		EBP	0.0140	0.0306	0.0237	0.0039
Standard Error	FIG	Direct	0.0013	0.0174	0.0078	0.0030
		EBP	0.0030	0.0071	0.0048	0.0010
	FIS	Direct	0.0002	0.0057	0.0023	0.0012
		EBP	0.0007	0.0021	0.0013	0.0004

**Fig 4:**
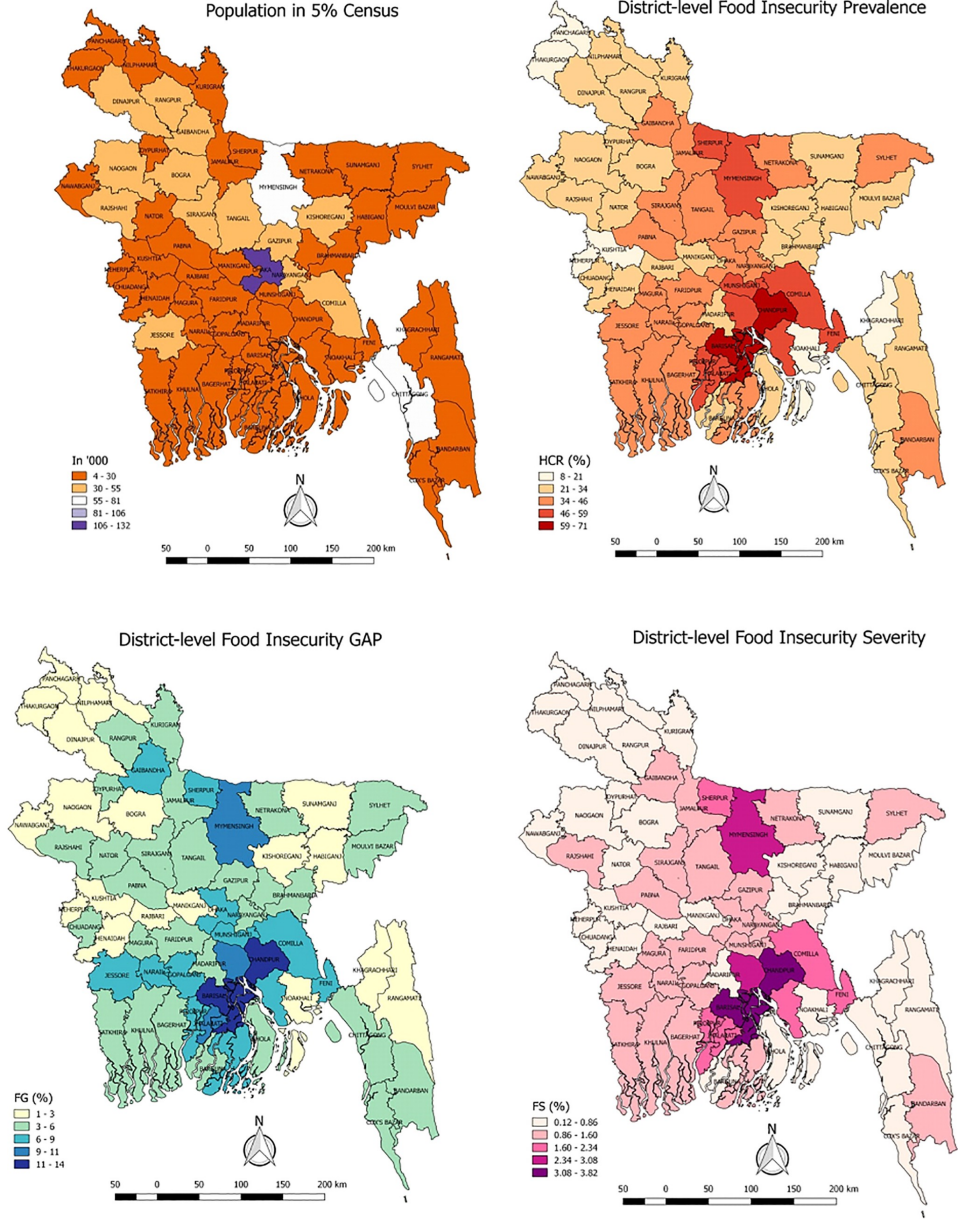
Cartograms of population in 5% census (upper left), estimated district level food insecurity prevalence (upper right), gap (lower left) and severity (lower right) in Bangladesh.

The FIP/HCR, FIG and FIS estimates calculated by the EBP method are presented in the cartograms of Fig 4. The maps show the spatial distribution of food insecurity indicators at district level. Darker regions of the maps correspond to the regions of high food insecurity. As the map demonstrates, food insecurity rates, intensity and severity are mainly concentrated more in the northern and southern parts of Bangladesh. For example, the Barisal and Chandpur districts in the southern part are found be the most vulnerable (FIP > 59%). Likewise, the districts under the recently announced Mymensingh division in the northern part of Bangladesh are found to be vulnerable in terms of all indicators. However, north-western part, north eastern part except Sylhet district, hill tracts area in south-eastern part except Bandarban district are found to be less vulnerable. The least vulnerable districts are Kushtia, Panchagarh, Meherpur, Thakurgaon, Noakhali, Khagrachhari, Nilphamari and Jhenaidah (FIP < 21%). Moreover, similar patterns are observed for the intensity and severity of food insecurity. The actual district-specific food insecurity estimates of all the indicators along with their percentage CVs and and 95 confidence intervals generated by the Direct and EBP methods are given in S1–S3 Appendices. The results reported in S1–S3 Appendices and maps in Fig 4 clearly show the degree of inequality with respect to distribution of food insecurity among the districts of Bangladesh. In particular, the maps show unequal distribution for all the three food insecurity indicators at district level in Bangladesh.

It is worth noting that the poverty indicators are calculated based on the Cost of Basic Needs (CBN) method in poverty mapping analysis, where the poverty line is calculated based on the household consumption expenditure for basic food and non-food items. The poverty line based on the cost for both items is referred to as upper poverty line, while the poverty line based on only the cost for the basket of basic food items is referred as lower poverty line. The quantities in the food basket are scaled according to the nutritional requirement of 2122 kcal per person per day, which is the food-insecurity line in this study. While the abovementioned upper poverty line is mainly used in the poverty mapping study. Consequently, the estimated food-insecurity prevalence based on the DCI method are not directly comparable with those head-count-rates estimated based on the CBN method. However, a relationship among these two types of indicators at district-level can provide a deeper insight to the policy makers. The most recent poverty mapping study based on the HIES 2010 and Census 2011 data indicates that the lowest (3.6%) and the highest (63.7%) HCRs were estimated for Kushtia (belongs to Khulna division) and Kurigram districts (belongs to Rangpur division) respectively (please see Table 5 of WB, BBS and WFP [15]). While this study based on food-intake measure indicates that estimated FIPs are 8.0% and 30.0% respectively for these two districts. For the capital Dhaka district, the estimated HCR was estimated as 15.7% while the FIP is estimated as 43.0% in this study. Similar opposite pattern is also observed for Rangpur (HCR: 42.0% and FIP: 27.6%) and Dhaka divisions (HCR: 30.5% and FIP: 40.5%). Such negative relationship between these two indicators is also observed for most of the districts of Dhaka and Rangpur divisions (please see Fig 4(B) and Zila Poverty Map of WB, BBS and WFP [15]). Some districts are highly vulnerable in terms of both HCR and FIP, as for example Barisal (FIP: 65.0%, HCR: 54.8%), Chandpur (FIP: 71.0%, HCR: 51.0%), Shariatpur (FIP: 58.0%, HCR: 52.6%), Sirajgonj (FIP: 38.0%, HCR: 38.7%), and Satkhira (FIP: 35.0%, HCR: 46.3%) districts. A similar positive relation is also observed for some of the least vulnerable districts like Noakhali (FIP: 19.0% and HCR: 9.6%) and Kushtia (FIP: 8.0%, HCR: 3.6%) districts. The negative relationship may come from either the varying poverty lines by strata or the same cut-off for the food-insecurity measure. These comparisons among poverty and food-insecurity indicators may help the policymakers to prioritize those districts highly vulnerable to both poverty and food-insecurity for proper food-aid intervention.

## Concluding remarks

Food insecurity maps are crucial for the allocation of funds by the governments and international organizations. Despite the importance, the local level food insecurity estimates in Bangladesh is lacking. The very latest available research conducted by the BBS in 2004, though as part of a poverty study, is now obsolete in terms of its use-effectiveness. To bridge this gap, this study aimed to estimate food insecurity prevalence at the district level in Bangladesh by using the latest available HIES 2010 dataset and the Census 2011. The reliable local level food insecurity indicators are estimated using the EBP method and as expected, the EBP estimates are found more reliable than direct estimates. For most of the districts, the reduction in CV is quite evident and the gains in efficiency of the EBP method tend to be larger for districts with smaller sample sizes. Finally, the generated district level cartograms of food insecurity prevalence, gap and severity indicates food insecurity indicators are mainly concentrated in the north and south areas of Bangladesh. In general, the degree of inequality with respect to the distribution of food insecure households among districts is quite high. Hence, the maps of this study help to show the districts with a relatively higher concentration of the food insecure people, which ultimately help the government, international organizations and policymakers for fund allocation and effective regional planning.

It is worth noting that the empirical performance of EBP and ELL methods cannot be directly compered because of differences in methodological settings of two methods. The empirical results may be compared by applying the EBP and the ELL based on a three-level model to accommodate both types of variation (cluster-specific and area-specific). However, it is tough to estimate both cluster-specific and area-specific consistent variance component simultaneously from the HIES 2010 or earlier data of Bangladesh due to insufficient number of clusters per district [24]. Further, when the target domains are at the very detailed level the ELL method is preferred to EBP due to computational simplicity of the ELL method, while if survey data contains information for most of the target domains, the EBP method can be preferred to the ELL method. Therefore, a comparative study can be done as a future research by implementing both the ELL and EBP method to the recent HIES 2016 [25] data (not yet fully available for the researcher) which covers many sub-districts, for estimating both district and sub-district level estimates of food insecurity in Bangladesh.

## Supporting information

S1 Data (CSV)

S1 Appendix District-wise values of direct and EBP estimates along with percentage coefficient of variation (CV,%) and 95% confidence interval (95% CI) of food insecurity prevalence (FIP/HCR) in Bangladesh. (DOCX)

S2 Appendix District-wise values of direct and EBP estimates along with percentage coefficient of variation (CV,%) and 95% confidence interval (95% CI) of food insecurity gap(FIG) in Bangladesh. (DOCX)

S3 Appendix District-wise values of direct and EBP estimates along with percentage coefficient of variation (CV,%) and 95% confidence interval (95% CI) of food insecurity severity (FIS) in Bangladesh. (DOCX)
