# Supplementary material for: Disaggregate level estimates and spatial mapping of food insecurity in Bangladesh by linking survey and census data
Source: PLoS One. 2020 Apr 10;15(4):e0230906. doi: 10.1371/journal.pone.0230906 (PMC7147775; doi:10.1371/journal.pone.0230906)
Supplement: S1 Appendix — (DOCX) [file pone.0230906.s002.docx]

**Table A1. District-wise values of direct and EBP estimates along with percentage coefficient of variation (CV,%) and 95 % confidence interval (95% CI) of food insecurity prevalence (FIP/HCR) in Bangladesh.**

| **District** | **Sample size** | **FIP/HCR** | | | | | | | |
| --- | --- | --- | --- | --- | --- | --- | --- | --- | --- |
|  |  | **Direct** | | | | **EBP** | | | |
|  |  | **Estimate** | **95% CI** | | **%CV** | **Estimate** | **95% CI** | | **%CV** |
|  |  |  | **Lower** | **Upper** |  |  | **Lower** | **Upper** |  |
| Barguna | 160 | 0.30 | 0.21 | 0.39 | 15.00 | 0.31 | 0.25 | 0.37 | 9.45 |
| Barisal | 200 | 0.62 | 0.50 | 0.73 | 9.29 | 0.65 | 0.60 | 0.70 | 3.98 |
| Bhola | 160 | 0.28 | 0.20 | 0.37 | 15.61 | 0.30 | 0.25 | 0.34 | 7.87 |
| Jhalokati | 160 | 0.50 | 0.39 | 0.62 | 11.42 | 0.55 | 0.50 | 0.59 | 4.32 |
| Patuakhali | 160 | 0.48 | 0.37 | 0.60 | 11.70 | 0.44 | 0.38 | 0.50 | 7.01 |
| Pirojpur | 140 | 0.50 | 0.37 | 0.62 | 12.52 | 0.53 | 0.47 | 0.59 | 5.53 |
| Bandarban | 140 | 0.30 | 0.20 | 0.39 | 16.23 | 0.34 | 0.30 | 0.39 | 6.71 |
| Brahmanbaria | 200 | 0.33 | 0.24 | 0.41 | 13.26 | 0.31 | 0.26 | 0.36 | 8.10 |
| Chandpur | 180 | 0.79 | 0.65 | 0.93 | 9.16 | 0.71 | 0.66 | 0.77 | 3.98 |
| Chittagong | 480 | 0.23 | 0.19 | 0.27 | 9.96 | 0.28 | 0.26 | 0.31 | 4.93 |
| Comilla | 300 | 0.55 | 0.46 | 0.64 | 8.28 | 0.54 | 0.50 | 0.57 | 3.55 |
| Cox's Bazar | 120 | 0.34 | 0.23 | 0.46 | 17.04 | 0.31 | 0.26 | 0.37 | 8.45 |
| Feni | 160 | 0.48 | 0.36 | 0.60 | 12.65 | 0.49 | 0.44 | 0.54 | 5.11 |
| Khagrachhari | 140 | 0.09 | 0.04 | 0.14 | 29.76 | 0.20 | 0.15 | 0.26 | 14.20 |
| Lakshmipur | 140 | 0.49 | 0.37 | 0.61 | 12.67 | 0.53 | 0.47 | 0.58 | 5.22 |
| Noakhali | 200 | 0.19 | 0.12 | 0.25 | 17.37 | 0.19 | 0.15 | 0.24 | 10.72 |
| Rangamati | 140 | 0.19 | 0.12 | 0.27 | 20.48 | 0.23 | 0.19 | 0.28 | 9.84 |
| Dhaka | 400 | 0.43 | 0.36 | 0.49 | 8.15 | 0.43 | 0.39 | 0.46 | 4.17 |
| Faridpur | 160 | 0.41 | 0.30 | 0.51 | 13.15 | 0.39 | 0.35 | 0.44 | 6.33 |
| Gazipur | 240 | 0.42 | 0.33 | 0.50 | 10.37 | 0.40 | 0.36 | 0.45 | 5.79 |
| Gopalganj | 160 | 0.44 | 0.33 | 0.55 | 12.76 | 0.46 | 0.41 | 0.51 | 5.70 |
| Jamalpur | 200 | 0.37 | 0.28 | 0.46 | 12.44 | 0.41 | 0.36 | 0.46 | 6.22 |
| Kishoregonj | 200 | 0.20 | 0.14 | 0.27 | 16.24 | 0.23 | 0.18 | 0.28 | 11.39 |
| Madaripur | 140 | 0.31 | 0.21 | 0.42 | 16.54 | 0.30 | 0.24 | 0.35 | 9.41 |
| Manikganj | 160 | 0.25 | 0.16 | 0.33 | 17.08 | 0.22 | 0.16 | 0.27 | 12.92 |
| Munshiganj | 120 | 0.55 | 0.41 | 0.70 | 13.50 | 0.47 | 0.41 | 0.52 | 6.33 |
| Mymensingh | 440 | 0.64 | 0.56 | 0.73 | 6.51 | 0.58 | 0.55 | 0.61 | 2.62 |
| Narayanganj | 240 | 0.38 | 0.30 | 0.46 | 11.22 | 0.39 | 0.35 | 0.43 | 5.28 |
| Narsingdi | 200 | 0.33 | 0.24 | 0.42 | 14.04 | 0.30 | 0.26 | 0.35 | 7.53 |
| Netrakona | 160 | 0.35 | 0.25 | 0.45 | 14.09 | 0.35 | 0.29 | 0.40 | 8.18 |
| Rajbari | 160 | 0.28 | 0.19 | 0.37 | 16.33 | 0.24 | 0.20 | 0.29 | 10.19 |
| Shariatpur | 120 | 0.66 | 0.50 | 0.82 | 12.37 | 0.58 | 0.53 | 0.64 | 4.75 |
| Sherpur | 160 | 0.44 | 0.33 | 0.55 | 12.52 | 0.52 | 0.46 | 0.57 | 5.34 |
| Tangail | 280 | 0.38 | 0.30 | 0.45 | 10.31 | 0.38 | 0.34 | 0.42 | 5.33 |
| Bagerhat | 160 | 0.43 | 0.32 | 0.53 | 12.91 | 0.40 | 0.36 | 0.45 | 5.86 |
| Chuadanga | 160 | 0.29 | 0.20 | 0.38 | 16.26 | 0.31 | 0.27 | 0.35 | 6.44 |
| Jessore | 240 | 0.37 | 0.29 | 0.46 | 11.14 | 0.46 | 0.42 | 0.50 | 4.41 |
| Jhenaidah | 180 | 0.20 | 0.13 | 0.27 | 17.53 | 0.21 | 0.17 | 0.26 | 10.55 |
| Khulna | 260 | 0.45 | 0.37 | 0.54 | 9.17 | 0.39 | 0.35 | 0.42 | 4.26 |
| Kushtia | 180 | 0.05 | 0.02 | 0.08 | 33.63 | 0.08 | 0.04 | 0.13 | 24.98 |
| Magura | 160 | 0.40 | 0.29 | 0.51 | 13.81 | 0.41 | 0.36 | 0.45 | 5.88 |
| Meherpur | 140 | 0.06 | 0.02 | 0.11 | 34.86 | 0.17 | 0.12 | 0.21 | 14.88 |
| Narail | 140 | 0.45 | 0.33 | 0.57 | 13.59 | 0.43 | 0.38 | 0.47 | 5.21 |
| Satkhira | 180 | 0.35 | 0.26 | 0.45 | 13.34 | 0.35 | 0.30 | 0.40 | 6.85 |
| Bogra | 240 | 0.25 | 0.18 | 0.31 | 13.50 | 0.28 | 0.24 | 0.31 | 6.49 |
| Joypurhat | 120 | 0.23 | 0.14 | 0.32 | 19.79 | 0.29 | 0.24 | 0.34 | 8.94 |
| Naogaon | 180 | 0.21 | 0.14 | 0.28 | 17.08 | 0.23 | 0.18 | 0.27 | 10.36 |
| Natore | 160 | 0.32 | 0.22 | 0.41 | 14.76 | 0.32 | 0.28 | 0.36 | 6.25 |
| Chapai Nababganj | 120 | 0.22 | 0.13 | 0.31 | 20.44 | 0.25 | 0.19 | 0.31 | 11.83 |
| Pabna | 200 | 0.36 | 0.27 | 0.45 | 12.62 | 0.38 | 0.34 | 0.43 | 5.59 |
| Rajshahi | 360 | 0.31 | 0.25 | 0.37 | 10.26 | 0.34 | 0.30 | 0.37 | 5.29 |
| Sirajganj | 200 | 0.38 | 0.29 | 0.47 | 11.99 | 0.38 | 0.34 | 0.42 | 5.57 |
| Dinajpur | 200 | 0.24 | 0.17 | 0.31 | 15.02 | 0.24 | 0.19 | 0.28 | 9.21 |
| Gaibandha | 160 | 0.43 | 0.32 | 0.54 | 12.59 | 0.45 | 0.40 | 0.50 | 5.76 |
| Kurigram | 180 | 0.31 | 0.23 | 0.40 | 14.33 | 0.30 | 0.26 | 0.34 | 6.77 |
| Lalmonirhat | 120 | 0.19 | 0.12 | 0.27 | 20.64 | 0.25 | 0.19 | 0.30 | 10.81 |
| Nilphamari | 140 | 0.25 | 0.16 | 0.34 | 17.81 | 0.21 | 0.16 | 0.27 | 12.55 |
| Panchagarh | 140 | 0.17 | 0.10 | 0.23 | 21.01 | 0.14 | 0.10 | 0.19 | 14.80 |
| Rangpur | 220 | 0.25 | 0.18 | 0.32 | 14.13 | 0.30 | 0.27 | 0.34 | 6.45 |
| Thakurgaon | 120 | 0.19 | 0.11 | 0.28 | 22.14 | 0.18 | 0.13 | 0.24 | 16.17 |
| Habiganj | 180 | 0.26 | 0.19 | 0.34 | 14.52 | 0.24 | 0.19 | 0.28 | 9.70 |
| Maulvibazar | 220 | 0.33 | 0.26 | 0.41 | 11.93 | 0.28 | 0.23 | 0.32 | 8.23 |
| Sunamganj | 220 | 0.26 | 0.19 | 0.33 | 13.39 | 0.24 | 0.20 | 0.28 | 9.45 |
| Sylhet | 240 | 0.29 | 0.22 | 0.36 | 12.31 | 0.36 | 0.33 | 0.40 | 5.15 |
