# Supplementary material for: Disaggregate level estimates and spatial mapping of food insecurity in Bangladesh by linking survey and census data
Source: PLoS One. 2020 Apr 10;15(4):e0230906. doi: 10.1371/journal.pone.0230906 (PMC7147775; doi:10.1371/journal.pone.0230906)
Supplement: S2 Appendix — (DOCX) [file pone.0230906.s003.docx]

**Table A2. District-wise values of Direct and EBP estimates along with percentage coefficient of variation (CV,%) and 95 % confidence interval (95% CI) of food insecurity gap(FIG) in Bangladesh.**

| **District** | **FIG** | | | | | | | |
| --- | --- | --- | --- | --- | --- | --- | --- | --- |
|  | **Direct** | | | | **EBP** | | | |
|  | **Estimate** | **95% CI** | | **%CV** | **Estimate** | **95% CI** | | **%CV** |
|  |  | **Lower** | **Upper** |  |  | **Lower** | **Upper** |  |
| Barguna | 0.039 | 0.025 | 0.052 | 17.67 | 0.040 | 0.027 | 0.053 | 16.41 |
| Barisal | 0.112 | 0.088 | 0.136 | 10.80 | 0.120 | 0.108 | 0.132 | 5.06 |
| Bhola | 0.039 | 0.024 | 0.054 | 19.49 | 0.037 | 0.025 | 0.049 | 16.32 |
| Jhalokati | 0.071 | 0.052 | 0.089 | 13.22 | 0.091 | 0.080 | 0.101 | 5.89 |
| Patuakhali | 0.065 | 0.047 | 0.083 | 14.13 | 0.063 | 0.049 | 0.077 | 11.26 |
| Pirojpur | 0.076 | 0.053 | 0.099 | 15.21 | 0.086 | 0.073 | 0.100 | 8.05 |
| Bandarban | 0.043 | 0.024 | 0.061 | 21.84 | 0.046 | 0.037 | 0.055 | 10.49 |
| Brahmanbaria | 0.035 | 0.024 | 0.046 | 16.14 | 0.041 | 0.030 | 0.051 | 13.01 |
| Chandpur | 0.167 | 0.133 | 0.201 | 10.40 | 0.141 | 0.130 | 0.153 | 4.11 |
| Chittagong | 0.026 | 0.020 | 0.032 | 12.71 | 0.036 | 0.030 | 0.043 | 9.19 |
| Comilla | 0.107 | 0.085 | 0.128 | 10.11 | 0.087 | 0.079 | 0.094 | 4.39 |
| Cox's Bazar | 0.042 | 0.025 | 0.058 | 20.25 | 0.041 | 0.031 | 0.052 | 13.38 |
| Feni | 0.069 | 0.048 | 0.091 | 15.72 | 0.076 | 0.066 | 0.085 | 6.27 |
| Khagrachhari | 0.009 | 0.002 | 0.016 | 39.86 | 0.023 | 0.012 | 0.035 | 25.43 |
| Lakshmipur | 0.081 | 0.057 | 0.106 | 15.53 | 0.084 | 0.074 | 0.094 | 6.05 |
| Noakhali | 0.023 | 0.014 | 0.032 | 19.84 | 0.022 | 0.013 | 0.030 | 19.79 |
| Rangamati | 0.023 | 0.008 | 0.038 | 32.79 | 0.028 | 0.019 | 0.036 | 15.27 |
| Dhaka | 0.068 | 0.055 | 0.081 | 9.61 | 0.064 | 0.056 | 0.072 | 6.58 |
| Faridpur | 0.061 | 0.041 | 0.081 | 16.44 | 0.055 | 0.045 | 0.065 | 9.35 |
| Gazipur | 0.059 | 0.044 | 0.074 | 12.75 | 0.058 | 0.049 | 0.068 | 8.36 |
| Gopalganj | 0.062 | 0.044 | 0.080 | 14.79 | 0.069 | 0.058 | 0.080 | 8.19 |
| Jamalpur | 0.049 | 0.034 | 0.063 | 15.13 | 0.058 | 0.048 | 0.069 | 9.12 |
| Kishoregonj | 0.030 | 0.018 | 0.042 | 20.15 | 0.027 | 0.016 | 0.038 | 21.55 |
| Madaripur | 0.046 | 0.028 | 0.064 | 19.79 | 0.037 | 0.025 | 0.049 | 16.51 |
| Manikganj | 0.026 | 0.015 | 0.037 | 21.29 | 0.025 | 0.013 | 0.036 | 23.41 |
| Munshiganj | 0.070 | 0.046 | 0.095 | 17.64 | 0.071 | 0.058 | 0.083 | 8.91 |
| Mymensingh | 0.102 | 0.086 | 0.118 | 7.96 | 0.098 | 0.092 | 0.104 | 3.14 |
| Narayanganj | 0.050 | 0.036 | 0.063 | 13.66 | 0.056 | 0.048 | 0.065 | 7.63 |
| Narsingdi | 0.043 | 0.028 | 0.057 | 17.09 | 0.039 | 0.029 | 0.049 | 13.18 |
| Netrakona | 0.046 | 0.028 | 0.064 | 20.22 | 0.046 | 0.033 | 0.059 | 14.17 |
| Rajbari | 0.029 | 0.016 | 0.042 | 22.99 | 0.029 | 0.019 | 0.038 | 16.32 |
| Shariatpur | 0.116 | 0.082 | 0.150 | 15.02 | 0.099 | 0.089 | 0.109 | 5.32 |
| Sherpur | 0.065 | 0.046 | 0.084 | 15.03 | 0.082 | 0.069 | 0.094 | 7.55 |
| Tangail | 0.046 | 0.033 | 0.058 | 14.10 | 0.052 | 0.045 | 0.060 | 7.54 |
| Bagerhat | 0.086 | 0.059 | 0.112 | 15.87 | 0.057 | 0.048 | 0.065 | 7.50 |
| Chuadanga | 0.033 | 0.020 | 0.046 | 20.24 | 0.039 | 0.033 | 0.046 | 8.21 |
| Jessore | 0.057 | 0.043 | 0.072 | 12.80 | 0.068 | 0.061 | 0.076 | 5.56 |
| Jhenaidah | 0.021 | 0.011 | 0.030 | 23.33 | 0.025 | 0.017 | 0.032 | 16.20 |
| Khulna | 0.076 | 0.059 | 0.093 | 11.31 | 0.055 | 0.049 | 0.060 | 5.43 |
| Kushtia | 0.004 | 0.000 | 0.008 | 44.65 | 0.008 | 0.001 | 0.015 | 44.74 |
| Magura | 0.056 | 0.038 | 0.073 | 15.95 | 0.058 | 0.049 | 0.066 | 7.54 |
| Meherpur | 0.003 | 0.001 | 0.006 | 39.75 | 0.018 | 0.009 | 0.027 | 26.20 |
| Narail | 0.066 | 0.045 | 0.086 | 16.01 | 0.062 | 0.054 | 0.070 | 6.60 |
| Satkhira | 0.056 | 0.039 | 0.073 | 15.54 | 0.046 | 0.038 | 0.055 | 9.31 |
| Bogra | 0.031 | 0.021 | 0.042 | 17.03 | 0.035 | 0.028 | 0.041 | 9.16 |
| Joypurhat | 0.028 | 0.014 | 0.042 | 24.92 | 0.037 | 0.028 | 0.046 | 12.07 |
| Naogaon | 0.022 | 0.013 | 0.032 | 22.12 | 0.026 | 0.018 | 0.035 | 16.48 |
| Natore | 0.043 | 0.027 | 0.058 | 18.80 | 0.042 | 0.035 | 0.049 | 8.18 |
| Chapai Nababganj | 0.034 | 0.018 | 0.050 | 23.88 | 0.031 | 0.018 | 0.043 | 20.49 |
| Pabna | 0.048 | 0.034 | 0.061 | 14.88 | 0.053 | 0.045 | 0.060 | 7.64 |
| Rajshahi | 0.045 | 0.033 | 0.056 | 13.05 | 0.045 | 0.038 | 0.052 | 7.47 |
| Sirajganj | 0.063 | 0.045 | 0.080 | 14.33 | 0.052 | 0.045 | 0.060 | 7.12 |
| Dinajpur | 0.033 | 0.021 | 0.045 | 18.33 | 0.028 | 0.020 | 0.036 | 14.51 |
| Gaibandha | 0.058 | 0.040 | 0.076 | 15.83 | 0.066 | 0.056 | 0.076 | 7.40 |
| Kurigram | 0.038 | 0.025 | 0.052 | 18.04 | 0.038 | 0.031 | 0.045 | 9.28 |
| Lalmonirhat | 0.022 | 0.012 | 0.032 | 23.02 | 0.029 | 0.020 | 0.039 | 16.10 |
| Nilphamari | 0.021 | 0.011 | 0.031 | 24.55 | 0.024 | 0.015 | 0.034 | 19.13 |
| Panchagarh | 0.022 | 0.012 | 0.033 | 24.30 | 0.015 | 0.008 | 0.022 | 23.48 |
| Rangpur | 0.032 | 0.021 | 0.044 | 17.81 | 0.039 | 0.032 | 0.046 | 9.26 |
| Thakurgaon | 0.019 | 0.010 | 0.029 | 25.60 | 0.020 | 0.010 | 0.030 | 25.83 |
| Habiganj | 0.035 | 0.022 | 0.048 | 19.11 | 0.028 | 0.018 | 0.038 | 18.23 |
| Maulvibazar | 0.049 | 0.035 | 0.064 | 14.93 | 0.035 | 0.026 | 0.044 | 12.80 |
| Sunamganj | 0.035 | 0.024 | 0.047 | 16.77 | 0.029 | 0.018 | 0.039 | 18.64 |
| Sylhet | 0.036 | 0.025 | 0.047 | 15.54 | 0.050 | 0.043 | 0.058 | 7.53 |
