# Supplementary material for: Disaggregate level estimates and spatial mapping of food insecurity in Bangladesh by linking survey and census data
Source: PLoS One. 2020 Apr 10;15(4):e0230906. doi: 10.1371/journal.pone.0230906 (PMC7147775; doi:10.1371/journal.pone.0230906)
Supplement: S3 Appendix — (DOCX) [file pone.0230906.s004.docx]

**Table A3. District-wise values of Direct and EBP estimates along with percentage coefficient of variation (CV,%) and 95 % confidence interval (95% CI) of food insecurity severity (FIS) in Bangladesh.**

| **District** | **FIS** | | | | | | | |
| --- | --- | --- | --- | --- | --- | --- | --- | --- |
|  | **Direct** | | | | **EBP** | | | |
|  | **Estimate** | **95% CI** | | **%CV** | **Estimate** | **95% CI** | |  |
|  |  | **Lower** | **Upper** |  |  | **Lower** | **Upper** | **%CV** |
| Barguna | 0.008 | 0.004 | 0.011 | 25.49 | 0.008 | 0.004 | 0.011 | 23.20 |
| Barisal | 0.029 | 0.021 | 0.036 | 13.37 | 0.031 | 0.027 | 0.034 | 5.84 |
| Bhola | 0.009 | 0.004 | 0.013 | 25.37 | 0.007 | 0.004 | 0.011 | 25.98 |
| Jhalokati | 0.015 | 0.010 | 0.020 | 17.85 | 0.022 | 0.019 | 0.025 | 7.08 |
| Patuakhali | 0.013 | 0.008 | 0.018 | 19.62 | 0.014 | 0.010 | 0.018 | 15.03 |
| Pirojpur | 0.018 | 0.010 | 0.026 | 23.56 | 0.020 | 0.016 | 0.024 | 9.96 |
| Bandarban | 0.011 | 0.003 | 0.019 | 35.57 | 0.010 | 0.007 | 0.012 | 14.24 |
| Brahmanbaria | 0.006 | 0.003 | 0.008 | 20.65 | 0.008 | 0.005 | 0.011 | 17.85 |
| Chandpur | 0.046 | 0.035 | 0.057 | 12.43 | 0.038 | 0.035 | 0.041 | 4.20 |
| Chittagong | 0.005 | 0.003 | 0.007 | 19.74 | 0.007 | 0.005 | 0.009 | 13.80 |
| Comilla | 0.030 | 0.022 | 0.038 | 13.51 | 0.021 | 0.019 | 0.022 | 5.03 |
| Cox's Bazar | 0.007 | 0.004 | 0.011 | 24.67 | 0.008 | 0.005 | 0.011 | 18.23 |
| Feni | 0.015 | 0.009 | 0.022 | 22.00 | 0.017 | 0.015 | 0.020 | 7.21 |
| Khagrachhari | 0.002 | 0.000 | 0.003 | 56.33 | 0.004 | 0.001 | 0.007 | 38.06 |
| Lakshmipur | 0.022 | 0.014 | 0.030 | 19.51 | 0.020 | 0.017 | 0.022 | 6.67 |
| Noakhali | 0.004 | 0.002 | 0.006 | 25.02 | 0.004 | 0.002 | 0.006 | 29.95 |
| Rangamati | 0.007 | 0.000 | 0.014 | 53.71 | 0.005 | 0.003 | 0.007 | 20.69 |
| Dhaka | 0.015 | 0.012 | 0.019 | 12.10 | 0.014 | 0.012 | 0.017 | 8.87 |
| Faridpur | 0.014 | 0.008 | 0.020 | 22.14 | 0.012 | 0.009 | 0.014 | 12.01 |
| Gazipur | 0.013 | 0.008 | 0.017 | 16.79 | 0.013 | 0.010 | 0.015 | 10.65 |
| Gopalganj | 0.013 | 0.008 | 0.017 | 17.94 | 0.015 | 0.012 | 0.018 | 10.37 |
| Jamalpur | 0.010 | 0.006 | 0.014 | 20.50 | 0.012 | 0.009 | 0.015 | 11.69 |
| Kishoregonj | 0.007 | 0.004 | 0.011 | 24.59 | 0.005 | 0.002 | 0.008 | 32.70 |
| Madaripur | 0.010 | 0.005 | 0.014 | 26.72 | 0.007 | 0.004 | 0.011 | 24.64 |
| Manikganj | 0.004 | 0.002 | 0.007 | 27.07 | 0.005 | 0.001 | 0.008 | 35.51 |
| Munshiganj | 0.015 | 0.008 | 0.022 | 23.55 | 0.016 | 0.012 | 0.019 | 10.97 |
| Mymensingh | 0.024 | 0.019 | 0.029 | 10.53 | 0.024 | 0.022 | 0.025 | 3.46 |
| Narayanganj | 0.010 | 0.006 | 0.013 | 18.13 | 0.012 | 0.010 | 0.014 | 9.71 |
| Narsingdi | 0.009 | 0.005 | 0.012 | 22.55 | 0.008 | 0.005 | 0.011 | 19.05 |
| Netrakona | 0.012 | 0.005 | 0.019 | 31.19 | 0.009 | 0.006 | 0.013 | 20.62 |
| Rajbari | 0.005 | 0.002 | 0.009 | 34.15 | 0.005 | 0.003 | 0.008 | 22.79 |
| Shariatpur | 0.029 | 0.019 | 0.039 | 18.35 | 0.024 | 0.021 | 0.027 | 5.73 |
| Sherpur | 0.015 | 0.009 | 0.020 | 18.37 | 0.019 | 0.016 | 0.022 | 9.25 |
| Tangail | 0.010 | 0.005 | 0.014 | 23.86 | 0.011 | 0.009 | 0.013 | 9.35 |
| Bagerhat | 0.026 | 0.015 | 0.037 | 21.32 | 0.012 | 0.010 | 0.014 | 8.59 |
| Chuadanga | 0.006 | 0.003 | 0.009 | 27.19 | 0.008 | 0.006 | 0.009 | 9.95 |
| Jessore | 0.013 | 0.009 | 0.017 | 16.20 | 0.015 | 0.013 | 0.017 | 6.40 |
| Jhenaidah | 0.004 | 0.001 | 0.007 | 43.06 | 0.004 | 0.002 | 0.006 | 22.11 |
| Khulna | 0.020 | 0.014 | 0.025 | 15.25 | 0.012 | 0.010 | 0.013 | 6.38 |
| Kushtia | 0.001 | 0.000 | 0.001 | 57.49 | 0.001 | 0.000 | 0.003 | 67.41 |
| Magura | 0.011 | 0.007 | 0.015 | 19.83 | 0.012 | 0.010 | 0.014 | 8.92 |
| Meherpur | 0.000 | 0.000 | 0.001 | 49.67 | 0.003 | 0.001 | 0.005 | 38.51 |
| Narail | 0.014 | 0.008 | 0.020 | 20.63 | 0.014 | 0.011 | 0.016 | 7.82 |
| Satkhira | 0.013 | 0.008 | 0.018 | 19.47 | 0.009 | 0.007 | 0.012 | 11.35 |
| Bogra | 0.006 | 0.004 | 0.009 | 20.62 | 0.007 | 0.005 | 0.008 | 11.57 |
| Joypurhat | 0.006 | 0.002 | 0.009 | 33.31 | 0.007 | 0.005 | 0.009 | 14.66 |
| Naogaon | 0.004 | 0.002 | 0.007 | 27.59 | 0.005 | 0.003 | 0.007 | 23.05 |
| Natore | 0.009 | 0.005 | 0.013 | 24.17 | 0.008 | 0.007 | 0.010 | 9.81 |
| Chapai Nababganj | 0.008 | 0.004 | 0.013 | 28.50 | 0.006 | 0.002 | 0.009 | 31.31 |
| Pabna | 0.010 | 0.006 | 0.013 | 19.79 | 0.011 | 0.009 | 0.013 | 9.30 |
| Rajshahi | 0.011 | 0.007 | 0.014 | 17.91 | 0.009 | 0.007 | 0.011 | 9.33 |
| Sirajganj | 0.016 | 0.010 | 0.021 | 17.69 | 0.011 | 0.009 | 0.013 | 8.33 |
| Dinajpur | 0.007 | 0.003 | 0.010 | 24.74 | 0.005 | 0.003 | 0.007 | 19.83 |
| Gaibandha | 0.012 | 0.007 | 0.017 | 20.71 | 0.015 | 0.012 | 0.017 | 8.84 |
| Kurigram | 0.008 | 0.004 | 0.012 | 24.80 | 0.008 | 0.006 | 0.009 | 11.57 |
| Lalmonirhat | 0.004 | 0.001 | 0.007 | 31.92 | 0.006 | 0.003 | 0.008 | 21.11 |
| Nilphamari | 0.003 | 0.001 | 0.005 | 31.89 | 0.005 | 0.002 | 0.007 | 25.89 |
| Panchagarh | 0.005 | 0.002 | 0.007 | 29.40 | 0.003 | 0.001 | 0.004 | 32.76 |
| Rangpur | 0.007 | 0.004 | 0.010 | 23.88 | 0.008 | 0.006 | 0.009 | 12.08 |
| Thakurgaon | 0.003 | 0.001 | 0.005 | 30.83 | 0.004 | 0.001 | 0.006 | 35.88 |
| Habiganj | 0.008 | 0.004 | 0.012 | 24.65 | 0.005 | 0.002 | 0.008 | 28.20 |
| Maulvibazar | 0.012 | 0.007 | 0.016 | 19.84 | 0.007 | 0.005 | 0.009 | 16.90 |
| Sunamganj | 0.007 | 0.004 | 0.011 | 22.94 | 0.005 | 0.002 | 0.009 | 29.31 |
| Sylhet | 0.007 | 0.004 | 0.010 | 20.24 | 0.011 | 0.009 | 0.012 | 9.70 |
